# 

**Published:** 1880-12

**Authors:** 


### CONTENTS:

PAGE.

ORIGINAL COMMUNICATIONS:

Migraine. By Thos. Lothrop, M. D., . 193

CLINICAL REPORTS:

Foreign Bodies in the Stomach. Reported by Chas. L. Dayton, M. D., . . . . 202
A Case of Puerperal Eclampsia. Reported by J. W. Keene, M. D., . . . . . 203

TRANSLATIONS:

Choroiditis in Relapsing Fever, by Dr. Julius Trompetter. From the German by Lucien Howe, M. D., . . . . . . . 205
The Innocuousness of Nitrous-Oxide Gas, and the Possibility of Producing Prolonged Anæsthesia by Means of this Gas. From the Dutch by P. W. Van Peyma, M. D., . . . . . . . . . 209

SELECTIONS:

On the Use of Olive Oil in Large Doses for Softening and Causing the Easy Expulsion of Biliary Calculi, . . . . . . 211
Transplantation of a Testicle from the Groin to the Scrotum, . . . . . . . 214
Rupture of Axillary Artery during Reduction of Luxation of the Humerus; Diffuse Traumatic Aneurism; Amputation at the Shoulder-Joint; Death; Remarks, . . 215
The Treatment of Rupture of the Uterus, . 217
A New Method of Applying Pressure in Traumatic Aneurism, . . . . . 218
Peptized Milk as Food for Infants and Invalids, . . . . . . . . 219
Pathognomonic Sign of Fracture of the Neck of the Femur, . . . . . . . 219
Primary and Secondary Amputation—which? 220
A Successful Case of Transfusion of Blood, 221
A New Remedy in Diphtheria, . . . 222
Homœopathy, . . . . . . 224
The Metric System in Medicine, . . . 225
Hernia in Children, . . . . . . 226
Progress in Treatment of Stricture of Urethra, 227
New Method of Arresting Gonorrhœa, . 229

EDITORIAL:

Pharmacal Failings, . . . . . 231
Are most Women Crazy? . . . . 235
Bad for the Temperance Cause, . . . 235
Registration under the New Medical Law, . 236

REVIEWS, . . . . . . . 236

